# The oral microbiota in oral squamous cell carcinoma: unravelling mechanisms and clinical potential

**DOI:** 10.1186/s12935-026-04414-z

**Published:** 2026-07-22

**Authors:** Amany Hany Mohamed Kamel, Nourhan El-Tantawy, Sarah Ahmed, Al-Hassan Soliman Wadan, Nermeen AbuBakr

**Affiliations:** 1 Oral Biology Department, Faculty of Dentistry, Cairo University, Cairo, Egypt; 2 Oral Biology Department, Faculty of Dentistry, Delta University, Mansoura, Egypt; 3 Ministry of Health and Population, Cairo, Egypt; 4 Oral Biology Department, Faculty of Dentistry, Galala University, Suez, Egypt

**Keywords:** Oral squamous cell carcinoma, Oral microbiome, Dysbiosis, *Porphyromonas gingivalis*, *Fusobacterium nucleatum*, *Treponema denticola*, Oncogenic viruses

## Abstract

Originating in the mucosal lining of the mouth, oral squamous cell carcinoma is the most common malignancy of the head and neck regions. Its pathogenesis is multifactorial, involving environmental exposures, genetic susceptibility, and lifestyle-related risk factors. Increasing evidence indicates that oral microbial dysbiosis contributes to the initiation and progression of OSCC. Under healthy conditions, the oral cavity harbors a diverse and functionally balanced microbial ecosystem that maintains mucosal integrity, supports immune homeostasis, and prevents colonization by pathogenic species. Disruption of this equilibrium, known as oral dysbiosis, is increasingly recognized as a key event in oral carcinogenesis. In OSCC, a shift toward pathogenic and pro-inflammatory microbial communities has been consistently observed, particularly involving periodontal bacteria such as *Porphyromonas gingivalis*,* Treponema denticola*, and *Fusobacterium nucleatum*. These organisms contribute to tumor progression by activating inflammatory and oncogenic signaling pathways, including NF-κB, STAT3, and PI3K/Akt; suppressing apoptosis; inducing epithelial–mesenchymal transition; and immune evasion, thereby creating a tumor-promoting microenvironment. In addition to bacterial dysbiosis, viral and fungal components of the oral microbiome may act as important cofactors in OSCC. High-risk Epstein–Barr virus (EBV) and human papillomavirus (HPV) have been implicated in disrupting tumor suppressor pathways, causing genomic instability, and modulating the immune response. Fungal species, particularly *Candida albicans*, may further contribute by producing carcinogenic metabolites and inducing chronic inflammation. This review provides an integrated overview of the oral microbiome in OSCC, focusing on the composition and protective roles of the core microbiota, factors influencing microbial stability, and mechanisms by which dysbiosis contributes to carcinogenesis. It also highlights the oral microbiome as a potential source of non-invasive biomarkers and discusses microbiome-targeted strategies, including prebiotics, probiotics, and postbiotics, as promising adjunctive approaches to restore microbial balance and reduce tumor-promoting inflammation.

## Background

Oral squamous cell carcinoma (OSCC) is a malignant epithelial neoplasm originating from the squamous cells of the oral mucosa, most commonly affecting the floor of the mouth, tongue, and inferior lip [1]. It may develop from *de novo* clinically normal mucosa or from premalignant lesions such as erythroplakia, leukoplakia, submucous fibrosis, and lichen planus [2]. According to global estimates for 2020, oral cancer accounted for over 377,713 new cases and nearly 177,757 deaths, with notable disparities in incidence and survival rates among different regions [3]. The disease predominantly affects males and is most commonly diagnosed in individuals between the fifth and sixth decades of life [4].

Despite advances in therapy, prognosis remains poor. While early-stage detection can yield five-year survival rates exceeding 80%, survival drops below 30% in advanced disease. Unfortunately, more than 60% of cases are diagnosed late, reflecting the asymptomatic nature of early disease, limited awareness, and diagnostic challenges in primary care [5]. Early lesions may present as red, white, or mixed mucosal patches or as non-healing extraction sockets; however, pain usually arises only in advanced stages. Primary care diagnostic accuracy remains suboptimal due to variable presentations and inadequate training. Biological heterogeneity further complicates outcomes; for example, even small tongue tumors can show aggressive invasion, underscoring the strong correlation between late diagnosis, advanced stage, and poor prognosis [6].

Prognosis is strongly influenced by clinical and pathological factors, including tumor thickness, depth of invasion, tumor-node-metastasis (TNM) stage, lymphovascular invasion, and cervical lymph node involvement. Neck metastasis is particularly critical, as it reduces survival by up to 50% [7]. Although surgical and adjuvant therapies continue to advance, prevention remains the most effective strategy. Primary prevention centers on modifying risk factors (tobacco, alcohol, betel quid), while secondary prevention emphasizes screening and timely referral for suspicious lesions [8].

The pathogenesis of OSCC arises from a complex interaction between environmental exposures (tobacco, alcohol, betel quid chewing, and radiation) and host factors, including genetic susceptibility and poor oral hygiene. Increasing evidence implicates chronic inflammation and oral microbiome dysbiosis as important contributors to carcinogenesis [1, 9]. According to the Expanded Human Oral Microbiome Database, the human oral cavity contains 774 bacterial species-level taxa. Among these, approximately 58% are officially named species, 26% are known only as uncultivated phylotypes identified by molecular sequencing, and 16% are unnamed but cultivated taxa [10].

Commensal microorganisms within the oral cavity form complex, multispecies biofilm communities that maintain oral and systemic health. Early colonizers such as *Streptococcus salivarius* establish the initial microbial ecosystem, followed by *Lactobacillus*, *Actinomyces*, *Neisseria*, and *Veillonella*, which co-adhere and interact to stabilize the microbial community. These microorganisms contribute to host homeostasis by regulating local environmental conditions, facilitating nutrient metabolism, and preventing pathogen colonization through competitive exclusion and maintenance of mucosal barriers [11]. Nitrate-reducing bacteria, including *Neisseria*, participate in oral nitrate metabolism, which can influence nitric oxide availability and support systemic cardiovascular and immune functions [12]. Beyond local effects, the oral microbiota influences systemic immunity; for example, certain *Lactobacillus* strains enhance interferon responses via dendritic and T cell interactions [13].

Although several reviews have discussed the compositional structure of the oral microbiome and its links with oral diseases, a comprehensive overview of its mechanistic and translational relevance to OSCC remains limited. This review provides an integrated perspective on how oral microbial dysbiosis contributes to OSCC development through interconnected mechanisms, including immune modulation, chronic inflammation, and microbial metabolite-mediated signaling. In addition, it highlights emerging evidence supporting the oral microbiome as a source of potential diagnostic biomarkers and as a target for preventive and therapeutic interventions. By linking microbial ecology with carcinogenic mechanisms and clinical applications, this review offers an updated framework for understanding the role of the oral microbiome in OSCC and identifies key directions for future research.

## Methods

A structured literature search was performed to identify studies on the oral microbiota and OSCC. Databases including PubMed, Scopus, and Web of Science were searched for articles published up to December 2025. Search terms included combinations of: “oral microbiome”, “oral microbiota”, “oral dysbiosis”, “oral squamous cell carcinoma”, “periodontal pathogens”, “*Fusobacterium nucleatum”*,* “Porphyromonas gingivalis”*,* “Treponema denticola”*, “oral cancer biomarkers”, “microbial biomarkers”, “probiotics”, and “oral microbiome therapy”. Original research, clinical studies, systematic reviews, and meta-analyses published in English were considered. Priority was given to studies addressing microbial composition, mechanisms linking dysbiosis to OSCC, microbial biomarkers, and microbiome-targeted therapies. Articles were screened by title and abstract, followed by full-text review; duplicates, studies lacking methodological detail, or unrelated work were excluded. Additional relevant references were identified through manual searches of cited literature.

### Core microbial communities and ecological balance

The Human Microbiome Project (HMP) initially defined the “core human microbiome” as genes shared across individuals within a body site and later expanded this to include common, temporal, ecological, functional, and host-adapted cores [14, 15]. The oral core microbiome encompasses the microbial community and its genomes within the oral cavity across these categories. Following the gut, it represents the second largest and most diverse microbiota, comprising over 700 bacterial species, as well as fungi, protozoa, and viruses that contribute to ecosystem stability. Minimal inter-individual variation supports using the oral core microbiome as a reference for microbial balance, or eubiosis [16].

Predominant bacterial taxa in healthy oral microbiota, identified by 16 S ribosomal DNA (16 S rDNA) analyses, include *Actinomycetota* (formerly *Actinobacteria;* includes the genera *Rothia*,* Actinomyces* and *Corynebacterium*), *Bacteroidota* (formerly *Bacteroidetes;* includes the genera *Prevotella*,* Porphyromonas* and *Capnocytophaga*), *Bacillota* (formerly *Firmicutes;* includes the genera *Granulicatella* and *Streptococcus*), *Fusobacteriota* (formerly *Fusobacteria;* includes the genus *Fusobacterium*), and *Pseudomonadota* (formerly *Proteobacteria;* includes the genera *Haemophilus and Neisseria*) [15].

In addition to bacteria, the oral mycobiome is dominated by the fungal genus *Candida*, particularly *Candida albicans* (*C. albicans*), along with other genera such as *Cladosporium*, *Aspergillus*, and *Malassezia*. The oral virome includes bacteriophages and eukaryotic viruses such as Human papillomavirus and Epstein-Barr virus, which may interact with bacterial communities and host immunity and have been implicated in oral carcinogenesis [17].

Distinct oral niches, such as saliva, mucosa, and tooth surfaces, harbor site-specific microbial communities. Saliva is rich in *Firmicutes*. The continuously renewing mucosal epithelium limits colonization, whereas the tongue’s papillae favor anaerobes such as *Porphyromonas*,* Actinomyces*,* Veillonella*, and *Prevotella*. Non-shedding tooth surfaces enable microbial accumulation and plaque formation, with supragingival and subgingival plaques differing due to oxygen and nutrient gradients. For instance, anaerobic bacteria, including *Fusobacterium*, *Actinomyces*, and *Veillonella*, are mainly found in subgingival plaque. The microbial communities on tooth surfaces are shaped by the teeth’s physiological and anatomical characteristics. Even different sites on the same tooth can host distinct microbial populations. Additionally, the oral microbiome changes at different life stages and dentition phases, highlighting the importance of defining the core microbiome in the context of host development and specific ecological niches [15].

**Fig. 1:**
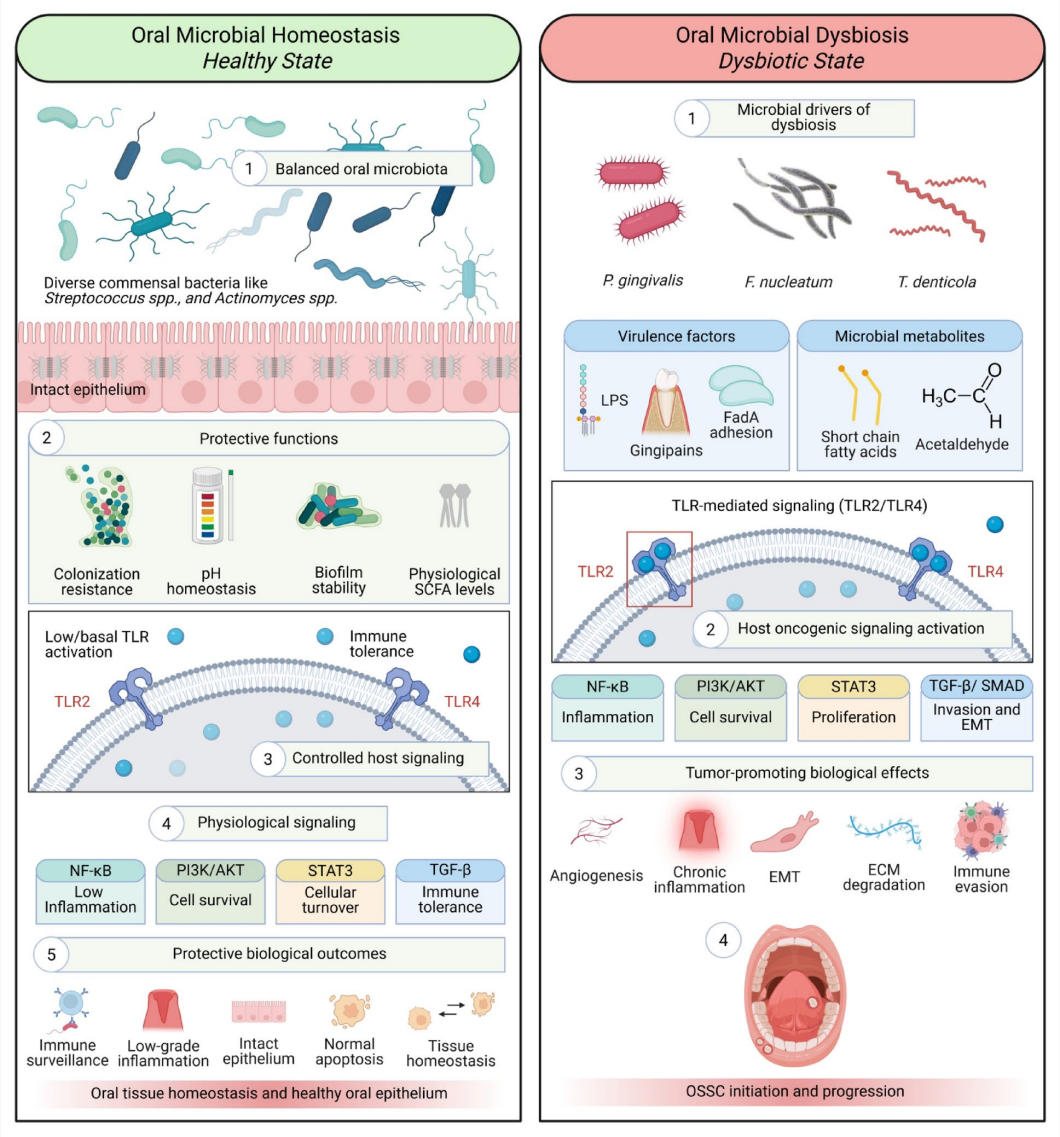
Oral microbial homeostasis versus dysbiosis in the development of OSCC. In a healthy state, a balanced and diverse oral microbiota maintains epithelial integrity, immune tolerance, and tissue homeostasis through controlled host signaling pathways. In contrast, microbial dysbiosis, characterized by enrichment of pathogenic taxa such as *P. gingivalis*, *F. nucleatum*, and *T. denticola*, promotes OSCC development via virulence factors and microbial metabolites that activate TLR-mediated oncogenic pathways, including NF-κB, PI3K/Akt, STAT3, and TGF-β signaling. These alterations drive chronic inflammation, immune evasion, EMT, extracellular matrix degradation, and angiogenesis, ultimately facilitating tumor initiation and progression. All figures have been created using BioRender.com

Ecological balance reflects a stable, mutually beneficial host-microbe relationship that supports systemic and oral health through niche-specific colonization, metabolic cooperation, and mutualistic interactions among microorganisms and the host. Salivary buffering systems, including bicarbonate and phosphate ions, maintain pH homeostasis and protect the oral environment from acidification. In parallel, nitrate-reducing bacteria within the oral microbiome convert dietary nitrate into nitrite and nitric oxide, which contribute to antimicrobial activity and modulation of host inflammatory responses. These combined mechanisms help maintain microbial equilibrium and reduce the risk of dysbiosis. Salivary buffering capacity and microbial adaptability further enhance ecosystem resilience, enabling recovery after disturbances such as antibiotic exposure or dietary changes [18] (Fig. 1).

### Protective roles of the oral microbiota

The physiological stability of the oral cavity relies upon its resident microbial communities for immune modulation, the structural maintenance of the mucosal epithelium, and resistance against pathogenic colonization. Under healthy conditions, commensal microorganisms interact with epithelial and immune cells to maintain a balanced microbial ecosystem (eubiosis). These host-microbe interactions contribute to immune priming by enhancing microbial recognition pathways and supporting controlled inflammatory responses that protect oral tissues from pathogenic invasion [19].

Commensal oral microorganisms also modulate the immune system by producing bioactive metabolites and signaling molecules. Metabolic products generated within oral biofilms, including short-chain fatty acids (SCFAs) and other microbial metabolites, can influence inflammatory signaling pathways, regulate immune cell activity, and promote mucosal immune tolerance. Through these interactions, the oral microbiota helps maintain an immune environment that balances antimicrobial defense with controlled inflammation [20, 21].

In addition to immune modulation, the oral microbiota helps maintain the physical and immunological barriers of the oral mucosa. Commensal microbes stimulate the production of secretory immunoglobulin A (sIgA), antimicrobial peptides, and anti-inflammatory cytokines that help preserve mucosal integrity and limit pathogen adhesion [22, 23]. Furthermore, many oral commensal species produce bacteriocins and other antimicrobial compounds that inhibit the growth of opportunistic pathogens and contribute to colonization resistance within oral biofilms [24]. Dysbiosis of this microbial balance can impair mucosal barrier function and promote chronic inflammation, creating a microenvironment that may increase susceptibility to OSCC and alter the oral microbial ecosystem.

### Factors affecting oral microbiome stability

#### 1. Host factors

Oral health results from the dynamic back-and-forth of host and environmental factors throughout life. The diversity and stability of the oral microbiota are shaped by host genetics, immunity, age, and lifestyle [25]. Host genetics accounts for at least 10% of microbial variance, as shown by metagenomic analyses and twin studies demonstrating greater similarity among monozygotic twins [18]. Saliva and gingival crevicular fluid provide essential nutrients and protective molecules, including peptides, glycoproteins, heme, and albumin, that sustain microbial communities and maintain equilibrium. Reduced salivary flow, as in xerostomia, promotes colonization by opportunistic microbes and dysbiosis [26].

#### 2. Environmental factors

Environmental exposures can significantly influence oral microbial composition. Indoor air pollutants, including mycotoxins, and contaminated food may suppress beneficial microbes, encourage pathogenic species, and impair immune defenses. Diet is a major determinant. High consumption of processed grains, refined saccharides, and animal fats promotes the proliferation of acid-producing and acid-tolerant bacteria, consequently driving the pathogenesis of cariogenic and periodontal diseases. By contrast, less industrialized diets, such as those of hunter-gatherer or agrarian populations, are associated with healthier oral microbiomes and lower disease prevalence [18].

#### 3. Lifestyle factors

Smoking disrupts oral microbial balance. Nicotine upregulates bacterial virulence genes and promotes biofilm formation via interactions with nicotinic acetylcholine receptors [27]. Tobacco smoke generates hypoxic conditions that favor anaerobes, increase free iron availability, and inhibit protective enzymes, such as oral peroxidases, resulting in dysbiosis with decreased *Pseudomonadota* and increased *Bacillota* and *Actinomycetota* [27, 28]. Oral hygiene is also critical. Poor hygiene allows plaque accumulation, which supports pathogens such as *Streptococcus mutans* and *F. nucleatum*, increasing the risk of caries, gingivitis, and periodontitis [27, 29]. Conversely, the prolonged administration of broad-spectrum antibiotics or oral antiseptics can cause a non-selective reduction of the oral microbiome, creating an ecological void that promotes opportunistic colonization [27].

#### 4. Systemic diseases

Systemic diseases can influence the stability of the oral microbiota by altering the local oral environment and host physiological conditions. For example, diabetes mellitus is associated with elevated glucose levels in gingival crevicular fluid and saliva, which can favor the growth of acidogenic and pathogenic microorganisms and alter the overall microbial composition. Diabetes is also associated with impaired immune responses and increased inflammatory mediators in the periodontal environment, creating conditions that facilitate the expansion of periodontopathogenic species [30].

Sjögren’s syndrome represents another condition that can significantly disrupt oral microbial balance. Changes in salivary composition and reduced salivary flow limit the natural cleansing and buffering functions of saliva, promoting the proliferation of opportunistic microorganisms such as *C. albicans* and cariogenic bacteria, including *Streptococcus mutans* [31].

Cardiovascular diseases have also been associated with alterations in oral microbial communities. Studies have reported shifts in the relative abundance of major bacterial phyla, including increased *Bacillota*/*Bacteroidota* ratio, reflecting changes in microbial ecology linked to systemic inflammatory states [32].

Together, these systemic conditions modify the oral environment by altering salivary composition, nutrient availability, and the inflammatory status, thereby disrupting microbial equilibrium and promoting oral dysbiosis.

### Microbial dysbiosis in OSCC

Oral dysbiosis is defined as an adverse alteration in the composition of the oral microbiome, wherein the established stability between host-compatible and pathogenic microorganisms is destabilized. Dysbiosis occurs when ecological disturbances promote the expansion of pathogenic taxa and the loss of protective microbial species, resulting in altered microbial composition and functional activity that may contribute to oral disease [19, 26]. The enrichment of periodontal pathogens, including *P. gingivalis*, *F. nucleatum*, and *T. denticola*, is a well-recognized example of oral dysbiosis. These microorganisms produce virulence factors that disrupt epithelial barriers, alter host signaling pathways, and stimulate chronic inflammation. Persistent colonization by such pathobionts creates a pro-inflammatory microenvironment, favoring epithelial proliferation, genomic instability, and tissue remodeling, processes that contribute to the initiation and progression of OSCC [18, 33].

Lipopolysaccharides (LPS), structural components of Gram-negative oral pathogens, further enhance the production of pro-inflammatory cytokines and activate inflammatory signaling pathways, thus sustaining a tumor-promoting microenvironment. Moreover, bacterial chemotaxis, flagellar assembly, and peptidases are enriched in the tumor tissue, confirming the role of inflammation in carcinogenesis [33].

High-throughput molecular techniques, specifically 16 S rRNA amplicon sequencing and whole-metagenome shotgun sequencing, bypass the limitations of traditional cultivation, enabling comprehensive taxonomic mapping of the oral microbiome across all microbial kingdoms [11]. Multi-omics approaches integrating metagenomic, transcriptomic, and metabolomic data have further improved understanding of microbial functional activities and their interactions with host carcinogenic pathways [34].

Host-microbe signaling at the mucosal barrier is mediated by specific pattern-recognition receptors, namely TLR2, TLR4, and NOD1/2, which are expressed by both healthy oral epithelium and OSCC cells to facilitate engagement with local pathobionts. Their activation triggers downstream signaling pathways, including NF-κB, leading to the production of pro-inflammatory cytokines, the activation of matrix metalloproteinases, and modulation of immune responses that contribute to tumor progression [35, 36]. Notably, microbial components, such as bacterial LPS, can induce PD-L1 expression in OSCC cells, and enrichment of bacteria, such as *Fusobacterium*, in oral tumors has been linked to elevated PD-L1 levels, thereby contributing to immune evasion and tumor progression [37, 38].

**Fig. 2:**
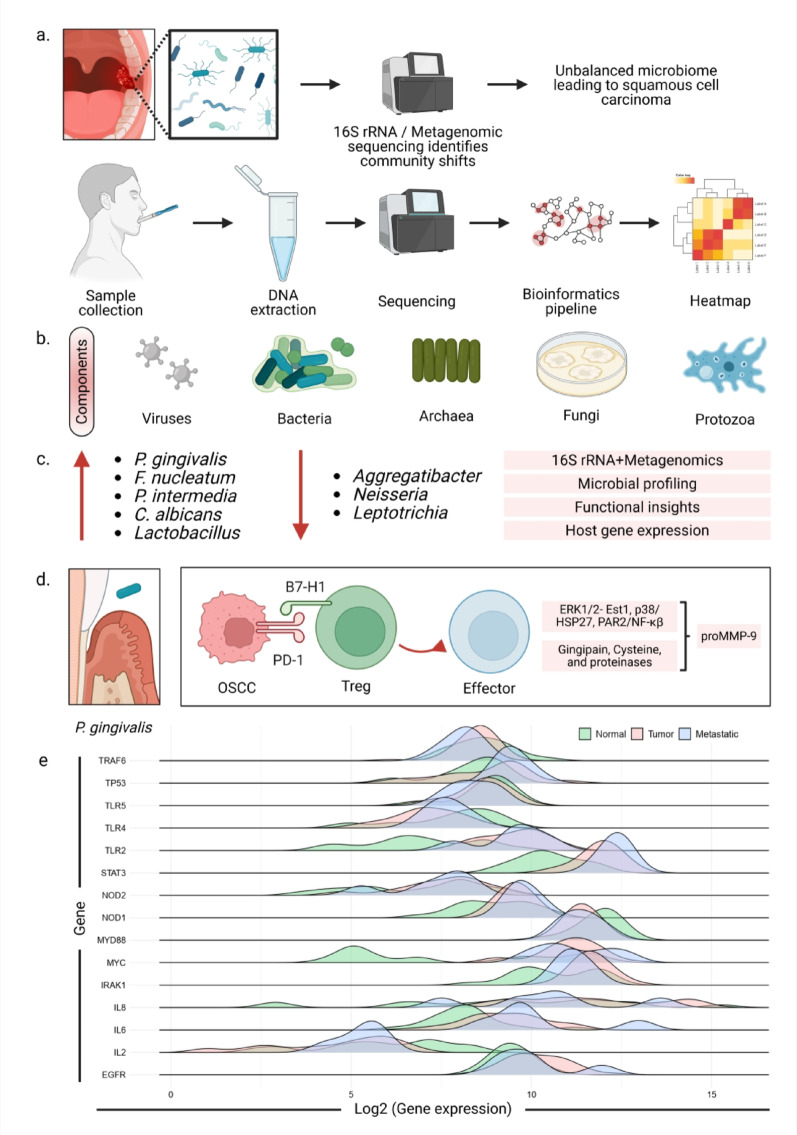
Overview of oral microbial dysbiosis and host-microbe interactions in OSCC. (**a**) Illustration of oral microbiome imbalance associated with OSCC and the workflow of culture-independent sequencing approaches, including sample collection, DNA extraction, sequencing, and bioinformatic analysis for microbial community profiling. (**b**) Major components of the oral microbiome, including viruses, bacteria, archaea, fungi, and protozoa, normally exist in ecological balance. (**c**) Representative microbial taxa altered in OSCC-associated dysbiosis. Pathogens such as *P. gingivalis*, *F. nucleatum*, *Prevotella intermedia*, and *C. albicans* are frequently enriched, whereas commensal genera including *Aggregatibacter*, *Neisseria*, and *Leptotrichia* are often reduced. (**d**) Host-microbe interactions at the epithelial interface, where microbial virulence factors activate pattern-recognition receptors and downstream pathways (e.g., ERK1/2 and NF-κB), promoting inflammation, immune modulation, and extracellular matrix degradation that facilitate tumor progression. (**e**) RNA-seq gene expression profiles derived from TNMplot comparing normal, tumor, and metastatic tissues in head and neck squamous cell carcinoma, highlighting genes involved in microbial sensing, inflammatory signaling, and oncogenic pathways. All figures have been created using BioRender.com

Gene expression patterns supporting these mechanisms have been revealed by analyzing publicly available RNA-sequencing datasets using TNMplot, which enables comparative visualization of transcriptomic profiles from normal and tumor tissues. These analyses show upregulation of genes involved in microbial sensing, inflammatory signaling, and oncogenic pathways, alongside reduced expression of tumor suppressor genes [39]. The main mechanisms linking oral dysbiosis to OSCC are summarized in (Figs. 1 and 2).

### Key taxa and mechanisms linking dysbiosis to OSCC

Several microbial taxa, notably *P. gingivalis*, *T. denticola*,* F. nucleatum*, and others, have been strongly implicated in OSCC pathogenesis (Table 1) [40].

**Table 1:** Key microorganisms implicated in OSCC and their mechanisms of action

Microorganism	Isolation source	Key mechanism(s)	Key molecular pathways / Effects	Primary evidence type / Strength
*Porphyromonas gingivalis* [42, 43, 48, 57]	Tumor tissue, saliva	Invades epithelial cells, suppresses apoptosis, and induces a pro-inflammatory microenvironment promoting proliferation and invasion	Activates PI3K/Akt, STAT3, and NF-κB signaling; upregulates MMP-9 and Bcl-2; increases IL-6 and IL-8 expression	Strong clinical association; animal and in vitro evidence; potential prognostic marker
*Fusobacterium nucleatum* [33, 50, 51, 81, 87, 88, 113]	Tumor tissue, saliva	Promotes chronic inflammation, immune evasion, EMT, and tumor cell proliferation; inhibits apoptosis via adhesin (FadA)	Activates E-cadherin/β-catenin and NF-κB signaling; induces IL-6, IL-8, and TNF-α release; enhances oncogenic transcription and anti-apoptotic pathways	Strong clinical association; supported by ex vivo mechanistic studies; potential diagnostic/prognostic biomarker
*Treponema denticola* [54–58]	Tumor tissue, saliva	Degrades host proteins via CTLP, promotes EMT, immune evasion, inflammation, and dysbiosis with other pathogens	Activates TLR-2/4 signaling and MMP pathways; promotes inflammatory responses and epithelial invasion	Ex vivo, animal model evidence and preliminary clinical evidence; enriched in OSCC tissues and saliva; implicated in immunomodulation and inflammatory pathways contributing to carcinogenesis.
*Prevotella intermedia* [15, 33, 34, 41, 88]	Tumor tissue, saliva	Enhances chronic inflammation and epithelial proliferation; associated with dysbiotic biofilms	Activates NF-κB and IL-6/STAT3 axis; induces COX-2 and MMP expression; promotes tumor progression	Preliminary clinical evidence; mechanistic support in vitro
*Peptostreptococcus* [80]	Tumor tissue	Alters immune responses and promotes oxidative stress and metabolic reprogramming	Activates NF-κB and p38 MAPK pathways; stimulates IL-8 secretion and DNA-damage-related carcinogenesis	Ex vivo / animal model mechanistic studies
*Capnocytophaga (e.g.*,* C. gingivalis)* [17, 113]	Tumor tissue, saliva	Associated with dysbiotic oral biofilms and increased abundance in OSCC samples; may contribute indirectly to tumor-associated inflammation	Linked to microbial community shifts rather than specific oncogenic pathways; limited evidence for direct signaling effects	Preliminary clinical association; observational microbiome studies without strong mechanistic validation
*Streptococcus spp. (e.g.*,* S. mitis*,* S. salivarius)* [41, 78, 87]	Saliva	Strain-dependent effects: some protective, others promote nitrosamine formation and inflammation	Nitrosamine formation; acetaldehyde production (strain-dependent properties among Streptococcus species); immune modulation and cytokine regulation; strain-specific effects on epithelial and immune responses.	Preliminary clinical evidence; strain-specific mechanistic studies
*Human papillomavirus* [59–61, 114]	Tumor tissue	Viral oncoproteins contribute to malignant transformation primarily in oropharyngeal SCC (OPSCC); limited role in OSCC	E6/E7 inactivate p53 and Rb pathways → cell-cycle dysregulation; p16 overexpression used as a surrogate marker (validated in OPSCC)	Strong evidence in OPSCC; limited and inconsistent evidence in OSCC
*Epstein–Barr virus (EBV)*[62, 63, 114]	Tumor tissue	Promotes epithelial proliferation and immune modulation	LMP-1 activates NF-κB and JAK/STAT signaling; induces pro-survival and inflammatory pathways	Preliminary clinical evidence; mechanistic in vitro support
*Candida albicans* [67–72, 74, 75]	Tumor tissue, saliva	Produces acetaldehyde and Candidalysin causing epithelial damage and inflammation	Induces oxidative stress, DNA double-strand breaks, and p53 activation; increases IL-1β and IL-8 expression	Strong clinical association; mechanistic in vitro evidence; implicated in malignant transformation

#### 1. Porphyromonas gingivalis

*P. gingivalis* is considered a representative periodontal pathogen in oral carcinogenesis. It promotes tumor survival and progression through multiple mechanisms: activation of NF-κB signaling pathways, PKB, Jak/Akt/STAT3, leading to upregulation of anti-apoptotic protein (Bcl-2), the apoptosis-inhibiting gene (Survivin), and oncogenes such as c-Myc [40–43].

Additionally, *P. gingivalis* modulates microRNA expression (e.g., miR-203) to suppress SOCS3, a cytokine signaling inhibitor, while secreting nucleoside diphosphate kinase that hydrolyzes extracellular ATP, disrupting ATP-mediated apoptosis. Together, these mechanisms synergistically enhance tumor cell survival [40, 42]. *P. gingivalis* produces LPS that interacts with the TLR4 receptor on human cells. This interaction initiates the NF-κB signaling pathway, enabling it to move into the nucleus and stimulate the production of pro-inflammatory cytokines such as interleukin 6 (IL-6), thereby establishing a microenvironment that promotes inflammation and tumor growth [41].

**Fig. 3:**
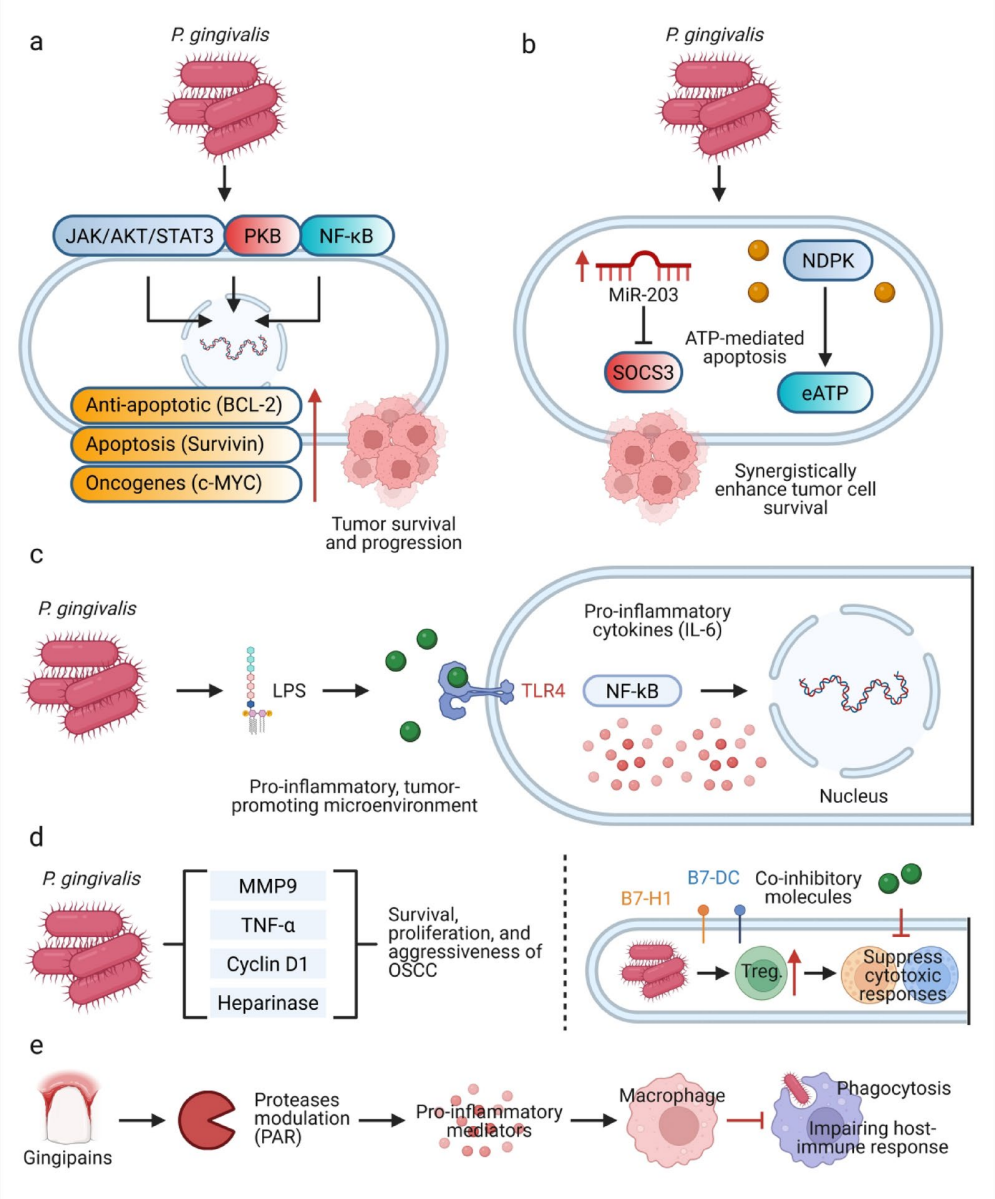
Overview of the multifaceted mechanisms by which *P. gingivalis* contributes to tumor survival and progression in OSCC. (**a**) depicts *P. gingivalis* activating key intracellular signaling pathways, including JAK/AKT/STAT3, PKB, and NF-κB, which leads to the upregulation of anti-apoptotic (BCL-2), apoptosis-inhibiting (Survivin), and oncogenic (c-MYC) genes in the cell nucleus, thereby promoting tumor survival. (**b**) shows the bacterium upregulating miR-203 to inhibit SOCS3, alongside other mechanisms involving nucleoside diphosphate kinase (NDPK) and extracellular ATP (eATP), which together are indicated to synergistically enhance tumor cell survival. (**c**) outlines how *P. gingivalis* LPS interacts with the cell-surface receptor TLR4 to activate NF-κB, triggering the production of pro-inflammatory cytokines like IL-6 and creating a pro-inflammatory, tumor-promoting microenvironment. (**d**) indicates that *P. gingivalis* induces the expression of tumor-promoting molecules such as MMP-9, TNF-α, Cyclin D1, and heparinase, while also fostering immune evasion through the induction of co-inhibitory molecules B7-H1 and B7-DC and Tregs that suppress cytotoxic immune responses. (**e**) demonstrates how gingipains from the bacterium modulate protease-activated receptors (PARs) to release pro-inflammatory mediators, which subsequently act on macrophages to inhibit phagocytosis, thus impairing the host immune response and leading to tissue destruction and degradation. All figures have been created using BioRender.com

*P. gingivalis* also produces bioactive molecules involved in the proliferation, survival, and aggressiveness of OSCC, such as cyclin D1, tumor necrosis factor-alpha (TNF-α), MMP-9, and heparinase, and promotes immune evasion by increasing co-inhibitory molecules B7-H1 and B7-DC, expanding Tregs that suppress cytotoxic responses [44, 45]. Gingipains, the cysteine proteases of *P. gingivalis*, modulate protease-activated receptors (PARs) signaling, induce pro-inflammatory mediators such as CXCL8, and impair host immune responses, including macrophage function and phagocytosis, thereby promoting tissue damage and disease progression (Fig. 3) [46, 47].

#### 2. Fusobacterium nucleatum

*F. nucleatum* contributes to OSCC by stimulating TLR-mediated IL-6/STAT3. It also induces key molecular markers of oral tumor growth, including heparinase, TNFα, cyclin D1, and MMP-9. It also upregulates IL-8, MMP-1, MMP-9, cell survival markers (JAK and MYC), and epithelial-mesenchymal transition (EMT) markers (TGF-β and ZEB1), driving tumor proliferation and promoting cell cycle progression [48, 49].

Metabolically, *F. nucleatum* accumulates at tumor margins, enhancing glycolysis and lactate production via GLUT1-dependent pathways, acidifying the microenvironment, supporting tumor invasiveness, and polarizing immunosuppressive M2-like macrophages [50].

Recent studies indicate that *F. nucleatum* contributes to OSCC progression through multiple mechanisms, by enhancing tumor cell proliferation by interacting with E-cadherin. It modulates the CDH1/β-catenin pathway, increasing nuclear β-catenin, thereby increasing Ki-67 expression and promoting cell growth [51].

**Fig. 4:**
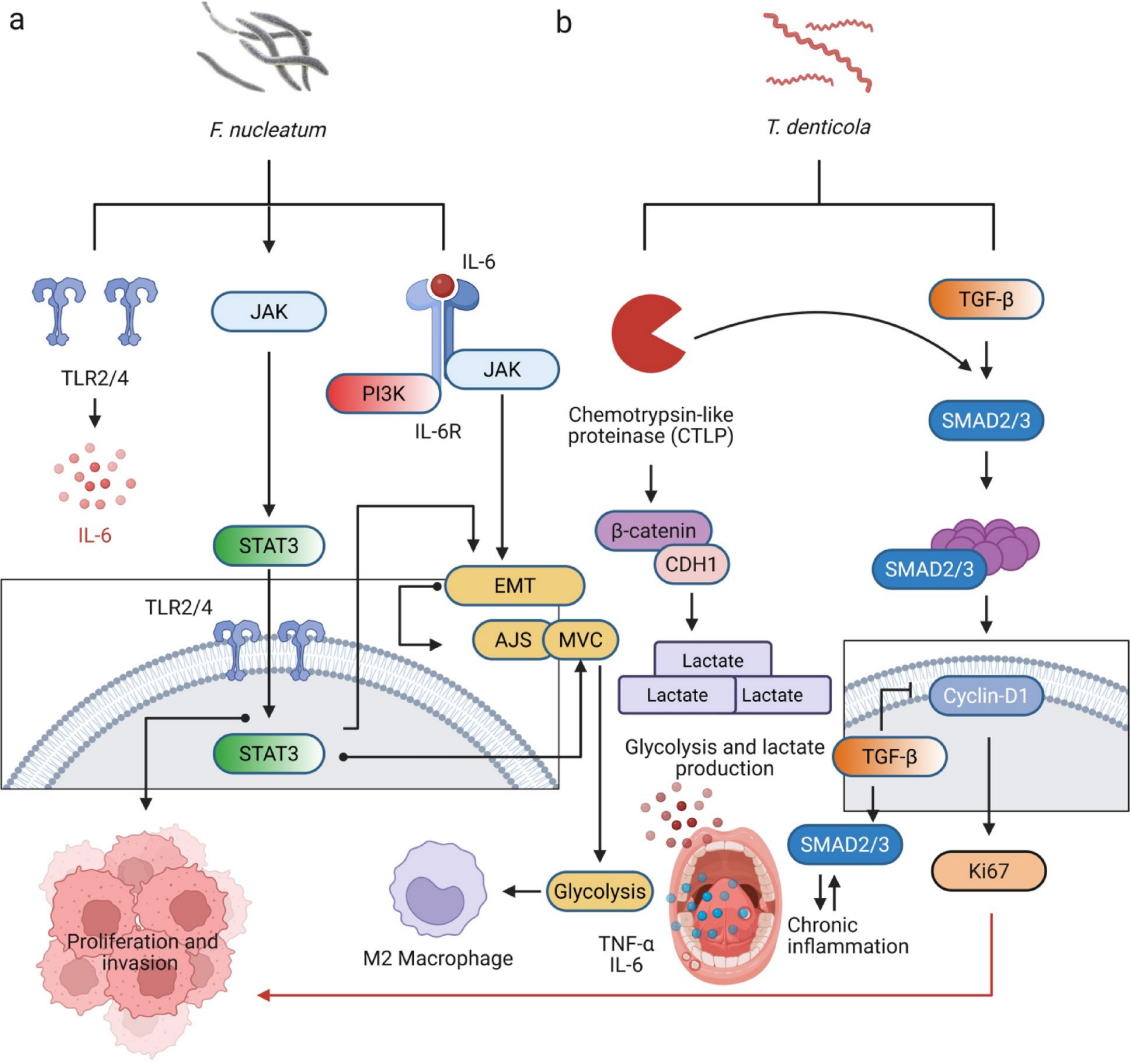
Overview of the mechanisms by which *F. nucleatum* and *T. denticola* contribute to OSCC progression. (**a**) *F. nucleatum* Signaling: *F. nucleatum* promotes tumor progression through the activation of TLR2/4 and IL-6 receptor (IL-6R) signaling. TLR2/4 activation induces IL-6 production, which binds to IL-6R to establish an autocrine signaling loop that activates the PI3K and JAK/STAT3 pathways. This amplified STAT3 activation promotes EMT—associated with adherens junctions (AJS) and microvascular changes (MVC)—as well as metabolic reprogramming (glycolysis) and M2 macrophage polarization. Ultimately, these interconnected pathways enhance tumor proliferation and invasion. (**b**) *T. denticola* Signaling: *T. denticola* contributes to carcinogenesis by secreting CTLP, which disrupts epithelial integrity via the β-catenin/CDH1 axis. This disruption enhances glycolysis and lactate production, fostering a tumor microenvironment characterized by chronic inflammation and driven by cytokines such as TNF-α and IL-6. Additionally, *T. denticola* activates the TGF-β/SMAD2/3 pathway. The translocation of the SMAD2/3 complex into the nucleus leads to Cyclin-D1 upregulation and increased expression of the cellular proliferation marker Ki-67, further driving tumor growth and invasion. All figures have been created using BioRender.com

The bacterium also promotes a pro-inflammatory tumor microenvironment, in part through NF-κB–mediated signaling, which supports tumor progression and survival [52]. Additionally, the *F. nucleatum* Fap2 protein can bind and attach to the inhibitory receptor TIGIT on T cells and natural killer cells, facilitating immune evasion and suppressing their anti-tumor activity [52, 53]. Collectively, these findings highlight the multifaceted role of *F. nucleatum* in promoting immune suppression, proliferation, and microenvironment remodeling in OSCC (Fig. 4).

#### 3. Treponema denticola

*T. denticola* promotes OSCC through proteolytic activity, cellular invasion, and oncogenic signaling. Its chymotrypsin-like proteinase (CTLP) degrades the extracellular matrix by activating MMP-9 and MMP-8, facilitating invasion. CTLP has also been linked to TLR-9 signaling, which correlates with greater invasion depth and more aggressive clinicopathological features. CTLP also disrupts immune surveillance by cleaving complement components, sustaining inflammation [54].

*T. denticola* has been shown to promote OSCC progression through activation of the TGF-β signaling pathway, leading to cell cycle progression, enhanced tumor cell proliferation, inhibition of apoptosis, and increased Ki-67 expression [55]. Its dentilisin can influence matrix remodeling and inflammatory processes by degrading cytokines such as TNF-α and IL-8, while also activating matrix metalloproteinases such as MMP-9 and MMP-8. In addition, it may counteract the inhibitory action of tissue inhibitors of metalloproteinases (TIMP-1 and TIMP-2), thereby creating conditions that favor epithelial invasion [56].

Emerging multi-omics and mechanistic evidence indicate that *T. denticola* promotes OSCC progression by activating TLR/MyD88 signaling, driving tumor cell migration, invasion, and inflammatory responses, partly through integrin/FAK signaling pathways [57, 58]. In parallel, *T. denticola* can produce hydrogen sulfide, which has been shown to promote OSCC cell proliferation by activating the COX-2/AKT/ERK1/2 signaling pathway (Fig. 4) [58].

### Viral content of tissue and saliva samples in OSCC

Viral involvement in OSCC has been extensively studied, particularly regarding the presence of viral DNA in tissue and saliva. Viral material is frequently detected in the tongue and palatine tonsils, likely due to their proximity to the oropharynx, a site of high viral exposure [17].

Human papillomavirus (HPV), especially high-risk genotypes HPV16 and HPV18, is a well-established carcinogen in oropharyngeal squamous cell carcinoma and a subset of head and neck squamous cell carcinomas, with emerging evidence regarding its role in OSCC [59]. HPV oncoproteins E6 and E7 disrupt cell cycle regulation by degrading tumor suppressors p53 and retinoblastoma protein (pRb), impair DNA repair, promote genomic instability, and inhibit apoptosis [60]. Overexpression of p16, a surrogate marker of HPV oncogenic activity, is commonly observed in HPV-associated oral cancers [61]. HPV is considered etiologically involved in approximately 3–4% of OSCC cases. However, reported HPV detection rates in OSCC vary substantially worldwide, ranging from about 6% to 58%, with high-risk types, particularly HPV-18 and HPV-16, being the most commonly identified [17].

Epstein-Barr virus (EBV), prevalent in human saliva, has been detected in 45% of OSCC cases [62]. EBV proteins, mRNA transcripts, and viral DNA (including the BamHIW and EBNA2 genomic regions) are infection biomarkers. Using these molecular indicators, cumulative evidence has demonstrated a consistent positive association between OSCC and EBV infection [63]. EBV has been associated with shifts in periodontal and peri-implant microbiota, including increased prevalence of pathogens such as *P. gingivalis*, suggesting synergistic microbial effects in tumor promotion [64]. EBV can trigger pathways associated with tumor development. The EGFR/PI3K/Akt signaling pathway is dysregulated in EBV-positive tumors. This pathway plays a fundamental role in cancer progression by enhancing NF-κB activity, suppressing pro-apoptotic proteins such as Bad and Bax, and thereby increasing the expression of genes that prevent apoptosis [62].

Emerging evidence links both HPV and EBV infections to oral microbial dysbiosis. Co-expression of HPV oncoproteins (E6, E7) and EBV latent membrane protein-1 (LMP-1) has been reported to cause DNA damage, potentially enhancing precancerous lesions progression [65]. While these observations support potential virus–bacteria interactions in tumor progression, the precise mechanistic links remain under investigation.

### Fungal content of OSCC tissue

Elevated levels of *Candida* species, particularly *C. albicans*, are frequently observed in OSCC and are associated with oral dysbiosis [66–70]. *C. albicans* is the predominant fungal species in OSCC patients, while non-*albicans* species such as *C. tropicalis*, *C. krusei*, *C. guilliermondii*, and *C. glabrata* are also detected in dysbiotic oral communities and are recognized as pathogenic fungi [67]. Normal commensal *C. albicans* can become pathogenic under conditions of compromised host defenses or altered local environments, such as in cancer patients or areas of mucosal damage. This pathogenic transition is linked to tissue invasion, chronic inflammation, and production of carcinogenic factors [71, 72].

Different *C. albicans* genotypes exhibit variable distribution patterns, with genotype C enriched in *Candida*-associated leukoplakia lesions. The presence of *Candida* in leukoplakia has been associated with increased epithelial dysplasia and a higher risk of malignant transformation, suggesting a potential contributory role of fungal superinfection in oral carcinogenesis [72, 73].

Recent multi-omics studies integrating metagenomic and metabolomic analyses have further highlighted *C. albicans’s* metabolic contribution to oral carcinogenesis. It was demonstrated that *C. albicans* can metabolize ethanol to acetaldehyde, a recognized carcinogen that induces DNA damage and mutagenesis in oral cells [41, 74].

*C. albicans* may also promote cancer through immune modulation, inflammation, and production of carcinogenic substances [41]. It generates nitrosamines, which damage DNA, impair DNA repair mechanisms, and increase mutation rates [41, 71]. It also stimulates proinflammatory cytokines (e.g., TNF-α, IL-8, IL-6) and activates T helper 17 (Th17) responses (IL-17, IL-23), which enhance tumor growth and angiogenesis and weaken antitumor immunity, ultimately supporting carcinogenesis [41].

*C. albicans* contribute to pathogenicity and cancer progression through several additional mechanisms. It produces virulence factors, such as acid aspartyl proteinases and the toxin candidalysin, while also suppressing protective molecules, such as β-defensins. Its presence is associated with increased expression of markers linked to malignancy, including p53, Ki-67, and COX-2, which are related to enhanced cell proliferation and inflammation. Moreover, *C. albicans* can interact with *P. gingivalis*, altering its gene expression and potentially enhancing bacterial infectivity. These combined effects are associated with greater tumor invasiveness and poorer prognosis [75].

### Oral microbiota as biomarkers in OSCC

Microbial dysbiosis in the oral cavity is increasingly recognized as a source of diagnostic and prognostic biomarkers for OSCC. Integrated microbial profiling studies have identified specific microbial signatures associated with OSCC. Genera such as *Gemella*,* Capnocytophaga*,* Streptococcus*,* Fusobacterium*,* Prevotella*,* Porphyromonas*,* Rothia*, and *C. albicans* have been consistently detected in tumor samples, highlighting their potential as recurrence monitoring and non-invasive biomarkers for OSCC detection [33, 34, 41]. A recent review of 82 studies published between 2016 and 2024 further highlights the growing research interest in the relationship between the oral microbiome and OSCC [76]. Ahmed et al. [77] demonstrated that saliva-derived DNA can detect somatic mutations in OSCC patients, emphasizing its diagnostic potential. Similarly, Lan et al. [78] reported increased abundance of *Gemella morbillorum*, *Streptococcus agalactiae*, and *Gemella haemolysans* in OSCC tissues, linking these microbes to cysteine- and methionine-metabolic pathways that may contribute to carcinogenesis. Tumor samples also frequently show elevated *Parvimonas* and reduced *Actinomyces*, with these microbial shifts correlating with tumor stage and disease progression [79]. Takahashi et al. [80] analyzed saliva from 60 Japanese OSCC patients and reported a higher prevalence of *Fusobacterium*,* Peptostreptococcus*,* Capnocytophaga*,* and Alloprevotella*,* while Rothia* and *Haemophilus* were less abundant compared with healthy controls. Another study has observed overrepresentation of *Pseudomonas aeruginosa* and *F. nucleatum* in tumor tissues [81]. Investigations in head and neck squamous cell carcinoma have revealed enrichment of several microbial taxa, including *P. gingivalis*, *Prevotella spp.*, *F. nucleatum*,* Streptococcus spp.*, *Capnocytophaga gingivalis*, and *Peptostreptococcus* species. These microorganisms are implicated in OSCC progression through mechanisms such as immune evasion, chronic inflammation, EMT, and the production of carcinogenic metabolites, including acetaldehyde and nitrosamines, as summarized in Table 1.

Metagenomic analyses further reveal functional differences in salivary microbiomes between OSCC, oral leukoplakia, and healthy controls, suggesting altered metabolic potential in disease states [78]. For example, *Rothia mucilaginosa*, enriched in leukoplakia lesions, can produce acetaldehyde from ethanol at levels that induce DNA damage and oxidative stress in oral epithelial cells [82]. Additionally, oral nitrate-reducing bacteria can convert dietary nitrates to nitrites and related metabolites, which under certain conditions can form potentially carcinogenic N-nitroso compounds [83]. Recent studies also highlight microbial activation of inflammatory and oncogenic signaling pathways, which are critical for OSCC initiation and progression.

Salivary biomarkers further complement microbial profiling and provide promising non-invasive tools for OSCC detection and monitoring. Several inflammatory cytokines, including TNF-α, IL-8, and IL-6, have been reported to be elevated in the saliva of OSCC patients, reflecting tumor-associated inflammation and the influence of microbial dysbiosis within the oral cavity [76, 84–86]. In addition to cytokines, microbial and metabolic biomarkers have also been proposed, including increased *F. nucleatum* abundance and its flagella, as well as microbial production of carcinogenic metabolites such as nitrosamines and acetaldehyde, which can induce oxidative stress, DNA damage, and mutagenesis in oral epithelial cells [33, 48, 81, 87]. Alterations in microbial composition, such as increased *Fusobacteriota* and decreased *Bacillota* and *Streptococcus* in saliva, have also been associated with OSCC and may serve as potential indicators for early detection or recurrence monitoring [88]. Furthermore, host molecular markers related to tumor progression, including p53 dysregulation, Ki-67 proliferation index, and activation of signaling pathways such as ERK/MAPK, have been linked to microbial-induced inflammation and metabolic alterations in OSCC tissues [89–91]. A summary of these salivary and tissue biomarkers associated with microbial dysbiosis, and their diagnostic or prognostic relevance, is presented in Table 2.

**Table 2:** Salivary, tissue and microbial biomarkers linked to microbial dysbiosis in OSCC

Biomarker	Type	Sample type	Associated microbes	Diagnostic/ prognostic/ monitoring use	Evidence level	Clinical relevance
IL-6 [41, 48, 84]	Pro-inflammatory cytokine	Saliva	*P. gingivalis*, *F. nucleatum* (dysbiosis)	Diagnosis	Moderate	Elevated salivary IL-6 reported in OSCC; promising non-invasive biomarker in multiple studies and meta-analyses; requires further validation.
IL-1β [41, 75]	Cytokine	Saliva / tissue	*F. nucleatum*, *C. albicans*,* P. gingivalis*	Monitoring inflammation	Moderate	Key mediator of inflammatory and Th17 responses; contributes to tumor-promoting microenvironment.
TNF-α [44, 48, 115, 116]	Cytokine	Saliva / tissue	*P. gingivalis*, *F. nucleatum*	Prognostic	Moderate	Chronic TNF-α elevation contributes to a pro-inflammatory tumor microenvironment and correlates with OSCC progression and severity.
C-reactive protein (CRP)[117]	Acute-phase protein	Serum (mainly) / saliva (less reliable	Mixed dysbiotic flora	Monitoring systemic inflammation	Moderate (Salivary CRP has limited clinical validation compared with serum CRP).	Reflects systemic inflammatory burden; associated with advanced stage and poorer prognosis in OSCC and head & neck cancers.
p16 protein [118]	Tumor suppressor surrogate	Tumor tissue	HPV (mainly HPV16/18)	Prognosis / diagnosis	High in OPSCC / Limited in OSCC	Validated surrogate marker for HPV-driven OPSCC; reduced specificity and limited utility in OSCC.
Candidalysin (ECE1 peptide) [119]	Fungal virulence factor	Tissue / epithelium	*C. albicans*	Mechanistic marker	Emerging	Mediates epithelial damage and inflammation; implicated in carcinogenic potential in experimental studies.
TRAIL [93]	Apoptosis ligand	OSCC cell lines (in vitro)	Microbial/ probiotic exposure	Preclinical therapeutic marker	Not clinical	Upregulated in experimental models after bacterial/probiotic exposure; induces apoptosis in vitro (not validated biomarker).
Nitrosamines/ N-nitroso compounds [41]	Carcinogenic metabolites	Saliva / mucosa	(*Candida* spp., oral microbiota)	Mechanistic / risk marker	Low	Microbial nitrosation may contribute to DNA damage and carcinogenesis; evidence mainly experimental.
Acetaldehyde (ACH) [82]	Oncometabolite	Saliva / mucosa	Oral bacteria (e.g., *Rothia mucilaginosa*)	Risk marker	Low/ moderate	Microbial acetaldehyde production induces oxidative stress and DNA damage in oral epithelium.
*F. nucleatum* abundance (DNA) [33, 50, 51, 81]	Microbial DNA marker	Saliva/ tissue	*F. nucleatum*	Diagnosis / Prognosis	Moderate	Increased abundance associated with OSCC and early recurrence risk; study-dependent variability.
*Firmicutes* / *Bacteroidetes* ratio [15, 32, 66, 120]	Microbial composition index	Saliva	*Broad microbiota*	Diagnosis (exploratory)	Low	Phylum-level shifts reported in OSCC cohorts; findings are heterogeneous across studies; not validated as a biomarker.
DNA methylation profile (CpG) [121]	Epigenetic marker	Tumor tissue	Microbial dysbiosis association	Prognosis / Mechanistic	Emerging	Microbiome-associated epigenetic alterations observed in integrative multi-omics studies.
SCFAs (short-chain fatty acids) [20, 113]	Microbial metabolites	Saliva /biofilms	Anaerobic commensals	Mechanistic	Emerging	Modulate immune responses, epithelial metabolism and signaling (e.g., via GPR41/43, histone deacetylase (HDAC) inhibition), their direct mechanistic role and clinical utility in OSCC are still under investigation.
p53 [60, 91, 122]	Tumor suppressor	Tissue	Dysbiosis-associated DNA damage	Prognostic	High	p53 alterations reflect DNA damage and inflammatory stress linked to microbial metabolites.
Ki-67 [89, 91]	Proliferation marker	Tissue	Microbial inflammatory signaling	Prognostic	Moderate–high	Correlates with tumor proliferation; influenced by inflammatory and microbial stimuli.
ERK1/2 / MAPK [38, 90]	Signaling protein	Tissue	Microbial dysbiosis association	Mechanistic/ Prognostic	Moderate	Microbe-induced activation promotes proliferation, invasion, and survival signaling.
GLUT1 / Lactate [50]	Metabolic marker	Tissue	Dysbiotic flora	Prognostic	Moderate	Tumor metabolic reprogramming with lactate accumulation contributes to immunosuppression and progression.

### Strategies to restore microbial balance in OSCC (Fig. 5)

#### 1. Probiotics

Probiotics are defined as viable microbial preparations that, upon administration in sufficient quantities, exert beneficial physiological effects on the host [92]. In OSCC, probiotics such as *Lactobacillus acidophilus* and *Lactobacillus Salivarius* has demonstrated antiproliferative and chemo-preventive effects in vitro and in animal models. These effects include induction of apoptosis in OSCC cells through upregulation of TNF-related apoptosis-inducing ligand (TRAIL) and the conversion of carcinogenic metabolites into non-toxic derivatives [93, 94]. Probiotics can also modulate host immune responses by enhancing anti-inflammatory cytokine production, promoting macrophage polarization, and activating antioxidant pathways, which may help restore microbial balance and reduce tumor-promoting inflammation [95–97]. 

**Fig. 5:**
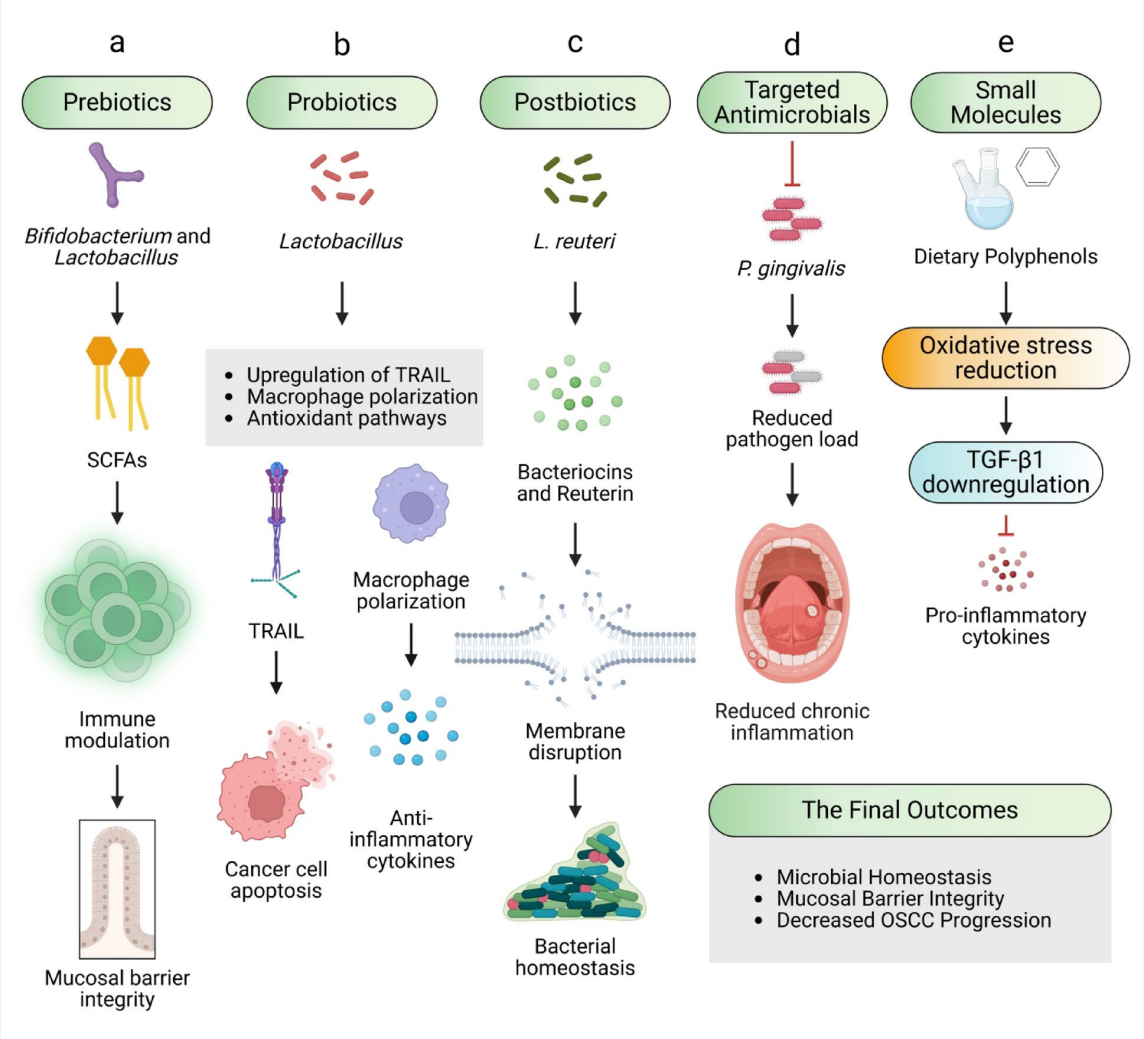
Therapeutic strategies to restore microbial balance and mitigate OSCC progression. The schematic illustrates five primary intervention pathways: (**a**) Prebiotics stimulate the proliferation of beneficial taxa (*Bifidobacterium* and *Lactobacillus*), leading to the production of SCFAs that drive immune modulation and reinforce mucosal barrier integrity. (**b**) Probiotics, such as *Lactobacillus* species, exert anti-tumorigenic effects by upregulating TRAIL to induce cancer cell apoptosis and by promoting macrophage polarization to increase anti-inflammatory cytokine production. (**c**) Postbiotics derived from *L. reuteri*, including bacteriocins and reuterin, maintain bacterial homeostasis through the targeted membrane disruption of opportunistic pathogens. (**d**) Targeted antimicrobials directly reduce the burden of carcinogenic pathogens like *P. gingivalis*, thereby attenuating chronic oral inflammation. (**e**) Small molecules, such as dietary polyphenols, reduce oxidative stress and downregulate TGF-β1 signaling, effectively suppressing the release of pro-inflammatory cytokines. Ultimately, these targeted, microbiome-modulating strategies converge to achieve microbial homeostasis, preserve mucosal barrier integrity, and decrease the overall progression of OSCC. All figures have been created using BioRender.com

#### 2. Prebiotics

Prebiotics function as indigestible dietary substrates that selectively promote the proliferation and metabolic output of commensal microorganisms. In the oral cavity, compounds such as fructooligosaccharides and inulin promote the proliferation of beneficial taxa, including *Lactobacillus* and *Bifidobacterium*, thereby supporting microbial homeostasis. These effects are associated with increased production of SCFAs, which modulate the immune response and may support epithelial barrier integrity [98–100]. In addition, several dietary compounds, including the polyphenols Epigallocatechin gallate and Curcumin, as well as the carotenoid Lycopene, exhibit prebiotic-like properties by modulating oral microbial communities and reducing oxidative stress and inflammatory signaling pathways such as TGF-β1. These compounds have shown therapeutic potential in precancerous oral lesions and may help reduce the risk of malignant progression [101–103].

#### 3. Postbiotics and antimicrobial peptides

Postbiotics encompass the biologically active byproducts secreted by probiotic strains, comprising a diverse array of metabolites such as SCFAs, bacteriocins, and functional proteins or peptides [104]. Bacteriocins are ribosomally synthesized antimicrobial peptides produced primarily by Gram-positive bacteria, particularly lactic acid bacteria. They typically exhibit a narrow inhibitory spectrum and act primarily by membrane disruption or pore formation, thereby limiting collateral effects on non-target microbial communities [105]. Other postbiotic metabolites, such as reuterin, exert broad antimicrobial and immunomodulatory effects that help maintain microbial homeostasis by suppressing pathogen overgrowth while supporting beneficial microbial populations [106]. In the context of OSCC-associated dysbiosis, these bioactive molecules may inhibit opportunistic pathogens enriched in oral cancer, while promoting restoration of mucosal barrier integrity and microbial balance [104, 107].

#### 4. Oral hygiene and restoration of microbial balance

Maintaining good oral hygiene remains a cornerstone in preventing dysbiosis-associated inflammation and OSCC progression. Mechanical plaque control, regular professional dental care, and appropriate antimicrobial interventions can significantly reduce colonization by pathogens strongly associated with oral carcinogenesis, including *P. gingivalis* and *F. nucleatum* [108]. These preventive measures help limit chronic inflammation, preserve epithelial barrier integrity, and support a balanced oral microbiome, thereby complementing microbiome-targeted therapeutic strategies.

### Challenges in precision targeting of oral microbiota in OSCC

#### 1. Microbial heterogeneity

Extreme variability in the oral microbiome between individuals and within different oral sites complicates the targeting of universal treatments. Disease stage and sample type further affect microbial profiles [109].

#### 2. Microbial resistance

As with antibiotics, microbes may develop resistance to targeted therapies, including phage therapy, through mechanisms such as receptor mutations and CRISPR-Cas systems, thereby reducing the long-term effectiveness of treatment. Additionally, misuse or overuse of antimicrobials can exacerbate dysbiosis and negatively impact oral microbial balance [110].

#### 3. Impact of conventional cancer treatments

Chemotherapy and radiotherapy markedly change the oral microbiota, frequently causing dysbiosis. This dysbiotic state is marked by a higher presence of opportunistic pathogens, which play a role in the development of oral mucositis. Emerging evidence indicates that interactions between the microbiome and cancer therapies influence treatment-related toxicity and clinical outcomes, highlighting the importance of pharmacomicrobiomics in optimizing therapeutic strategies [111].

#### 4. Need for advanced analytical tools

Reliable microbiological biomarkers for OSCC risk, progression, and response remain elusive. Improved sequencing methods and artificial intelligence are being explored to address this gap [112].

### Limitations and gaps in research on the relationship between OSCC and oral microbiota

Several important limitations constrain research on the oral microbiome in OSCC. Small cohort sizes reduce statistical significance and power, increasing the likelihood of false-negative or false-positive associations and limiting the generalizability of findings across diverse populations. Sampling variability is another key challenge: saliva, oral rinses, and tumor biopsies capture distinct microbial communities, with saliva reflecting transient populations and biopsies providing direct insight into the tumor microenvironment. Moreover, many studies fail to adequately account for confounding factors such as alcohol consumption, smoking, diet, oral hygiene, treatment history, and disease stage, all of which profoundly influence microbial composition. Technical inconsistencies, including bioinformatic pipelines, sequencing platforms, and DNA extraction methods, further complicate cross-study comparisons [27, 112]. Furthermore, most available studies are cross-sectional, limiting the ability to determine temporal relationships and causal associations between microbial alterations and OSCC development. Well-designed prospective longitudinal studies are therefore needed to establish causality and to characterize microbiome dynamics throughout disease initiation, progression, and treatment.

### Future perspectives

Future priorities in oral microbiome research in OSCC include identifying reliable microbial biomarkers for early detection, clarifying interactions between the microbiota and host immunity, and exploring how age-related dysbiosis contributes to oral carcinogenesis. Advanced experimental models, such as organoids and “organ-on-a-chip” platforms, offer promising tools for studying host-microbe-cancer interactions in a controlled environment, allowing mechanistic insights that cannot be captured in conventional in vitro or animal studies. Furthermore, the development and adoption of standardized protocols for the generation, culture, and analysis of these models will be essential to improve reproducibility, facilitate cross-study comparisons, and accelerate translation of research findings into clinical applications.

Microbiota-targeted interventions, including postbiotics, probiotics, microbiota transplantation, prebiotics, and phage therapy, have the potential to restore oral eubiosis and improve treatment outcomes in OSCC. However, these approaches require well-designed clinical trials and rigorous preclinical validation to confirm efficacy and safety. Ethical and technical considerations, such as invasive sampling, consent for biobanking, and regulatory requirements, must be addressed before clinical implementation.

A summary of key challenges, research gaps, and future directions in oral microbiota-targeted strategies for OSCC is presented in Table 3. This table highlights microbial heterogeneity, resistance, therapy impact, lack of validated biomarkers, oncogenic mechanisms, causality ambiguity, sample/population variation, ethical challenges, and the need for intervention validation. It serves as a roadmap for prioritizing research and designing targeted, effective, and safe microbiome-based interventions in OSCC management.

**Table 3:** Challenges, research gaps, and future directions in oral microbiota and OSCC

Aspect	Key issues / Findings	Implications	Future directions
Microbial heterogeneity [123]	Interindividual and site-specific variability in microbiota, influenced by disease stage and sampling method.	Hinders standardization and universal microbiome-targeted therapies.	Develop site-specific diagnostic models; use machine learning for personalized profiling.
Microbial resistance [124]	Resistance to targeted therapies (e.g., phage therapy) worsens by antimicrobial misuse.	Reduces the long-term efficacy of microbial interventions.	Explore phage engineering, combination therapies, and stewardship guidelines.
Cancer therapy impact [125]	Chemotherapy/radiotherapy disrupts oral microbiota, causing mucositis and dysbiosis.	Increases the risk of secondary infections and reduces treatment efficacy.	Integrate microbiome-preserving strategies during cancer treatment.
Lack of biomarkers [112]	Absence of validated microbial markers for OSCC risk, stage, or recurrence.	Limits early detection and monitoring.	Apply multi-omics and artificial intelligence to identify robust biomarkers.
Microbial oncogenic mechanisms [51 ,52]	Bacteria (e.g., *F. nucleatum*) activate NF-κB, Wnt/β-catenin, and TLR pathways.	Promotes EMT, stemness, inflammation, and immune evasion.	Target microbiota-driven pathways in therapy development.
Causality ambiguity [66]	Current evidence is predominantly derived from cross-sectional and association-based studies, making it unclear whether microbial dysbiosis is a driver or a consequence of OSCC.	Limits causal inference and challenges the establishment of microbiota as true etiological factors in carcinogenesis.	Conduct longitudinal, prospective, and mechanistic studies to clarify temporal dynamics and establish causal relationships between microbiome alterations and OSCC development.
Sample/population variation [123, 126]	Results vary due to geography, lifestyle, sampling technique, and sequencing method.	Reduces reproducibility and comparability across studies.	Standardize protocols; increase global and multi-ethnic cohorts.
Ethical challenges [123]	Concerns over invasive sampling, consent, and privacy in microbiome biobanking.	Hinders inclusion of vulnerable populations (e.g., elderly).	Establish ethical frameworks for microbiome-based diagnostics and interventions.
Intervention validation [112]	Limited clinical trials on probiotics, postbiotics, or microbiota modulation in OSCC.	Restricts clinical adoption of microbiome-based therapies.	Conduct large-scale randomized controlled trials (RCTs) to assess safety, efficacy, and sustainability.

## Conclusion

Oral microbial dysbiosis is increasingly acknowledged as both a factor that contributes to the development and progression of OSCC, and as a result of tumor-related changes in the oral microenvironment. Current evidence indicates a two-way relationship in which dysbiosis, marked by increased pathogenic bacteria such as *P. gingivalis*, *T. denticola*,* and F. nucleatum*, drives chronic inflammation, modulates the immune response, leads to metabolic reprogramming, and activates oncogenic signaling pathways. These processes collectively support tumor development and progression. Conversely, OSCC-associated alterations in tissue architecture, immune responses, and local metabolic conditions further disrupt microbial homeostasis, reinforcing a pro-carcinogenic environment. Beyond bacteria, viral and fungal components of the oral microbiome, including human papillomavirus, Epstein–Barr virus, and *C. albicans*, may act as additional cofactors that amplify inflammatory and genotoxic processes, contributing to malignant transformation. Collectively, these findings highlight the oral microbiome as an integrated ecological system actively involved in OSCC pathobiology rather than a passive bystander. Importantly, emerging evidence suggests that microbial signatures and associated host inflammatory mediators may serve as promising non-invasive biomarkers for early detection, prognosis, and disease monitoring. In parallel, microbiome-targeted strategies, such as probiotics, prebiotics, postbiotics, and optimized oral hygiene practices, represent potential adjunctive approaches for restoring microbial balance and reducing tumor-promoting inflammation. However, current evidence is limited by methodological heterogeneity, sampling variability, and lack of longitudinal data, and causality has not yet been fully established. Future research should focus on multi-omics, mechanistic, and longitudinal studies to validate microbial signatures and clarify host–microbe interactions. Integrating microbiome data with host molecular profiling may ultimately support the development of personalized preventive and therapeutic strategies, improving early detection and clinical outcomes in OSCC.

## Data Availability

No datasets were generated or analysed during the current study.

## Abbreviations

Akt: Protein Kinase B
CDH1: Cadherin-1, also known as E-cadherin
c-Myc: Cellular myelocytomatosis oncogene
COX-2: Cyclooxygenase-2
CTLP: Chymotrypsin-like proteinase
EMT: Epithelial-mesenchymal transition
ERK: Extracellular signal-regulated kinase
EBV: Epstein–Barr virus
FadA: Fusobacterium adhesin A
GLUT1: Glucose transporter type 1
HPV: Human papillomavirus
IL: Interleukin
JAK: Janus kinase
Ki-67: Marker of cellular proliferation
LMP-1: Latent membrane protein-1
LPS: Lipopolysaccharides
MAPK: Mitogen-activated protein kinase
MMP: Matrix metalloproteinase
MyD88: Myeloid differentiation primary response 88
NF-κB: Nuclear Factor Kappa-B
OPSCC: Oropharyngeal squamous cell carcinoma
OSCC: Oral squamous cell carcinoma
PI3K: Phosphoinositide 3-Kinase
Rb: Retinoblastoma
SCFAs: Short-chain fatty acids
SOCS3: Suppressor of cytokine signaling 3
STAT3: Signal transducer and activator of transcription 3
TGF-β: Transforming growth factor-beta
Th17: T helper 17
TLR: toll-like receptor
TNF-α: Tumor necrosis factor-alpha
TRAIL: TNF-related apoptosis-inducing ligand
Tregs: regulatory T cells
VEGF: Vascular endothelial growth factor
Wnt: Wingless-type MMTV integration site family
